# Improving the Transparency and Replicability of Consensus Methods: Respiratory Medicine as a Case Example

**DOI:** 10.2147/POR.S478163

**Published:** 2024-10-15

**Authors:** Mark J Rolfe, Christopher C Winchester, Alison Chisholm, David B Price

**Affiliations:** 1 Oxford PharmaGenesis, Oxford, UK; 2 Green Templeton College, University of Oxford, Oxford, UK; 3 Observational and Pragmatic Research Institute, Singapore, Singapore; 4 Centre of Academic Primary Care, Division of Applied Health Sciences, University of Aberdeen, Aberdeen, UK

Pragmatic and Observational Research strongly encourages all authors reporting the results of studies using consensus methods to follow the ACcurate COnsensus Reporting Document (ACCORD) guideline^1^,^2^ to ensure consistent, transparent reporting with sufficient detail to allow study replication of consensus methods and informed interpretation of the results.

Consensus studies play a critical role in biomedicine, supporting pragmatic decision-making in areas in which the existing evidence is equivocal, limited, absent, or still developing.^1^,^3–6^ Consensus approaches generally use iterative processes to synthesize expert opinions so that outputs are based on the collective knowledge and expertise of participants.^7^,^8^ Formal methodologies exist to guide and optimize the process of achieving consensus,^7^ such as the Delphi method,^9^,^10^ nominal group technique (NGT),^11^ RAND/UCLA Appropriateness Method,^12^ and structured consensus meetings.^13^ These established consensus methods differ in terms of anonymity, group size, and the nature of participant interactions (eg, face-to-face vs virtual meetings, or no meetings), allowing the appropriate method to be selected in the context of specific research questions and settings. Regardless of any differences, all formal methodologies generally aim to engage relevant stakeholders, encourage equitable contributions from participants, and minimize potential sources of bias.^7^

Consensus processes, especially the Delphi method, are well established in biomedicine and widely published in the scientific literature. A targeted search of the MEDLINE bibliographic database (conducted July 16, 2024) for publications on consensus conferences, NGT, Delphi, or RAND/UCLA methods identified 27,235 publications since 1946, of which 6117 (22%) were published in the period January 1, 2020 to July 16, 2024 (see Figure 1). The utility and adaptability of consensus methods were apparent when the identified publications were considered by type and across therapy areas. For example, 2048 of the consensus studies published since 2020 relate to respiratory medicine, which is a therapy area that spans acute and chronic conditions and communicable and noncommunicable diseases, affects individuals across the age spectrum, and contributes significantly to global mortality.^14^ Within respiratory medicine, it was evident from the literature that consensus methodologies have been used to: facilitate disease diagnosis and management;^15^,^16^ assess treatment choice (including delivery method, dosing, and duration);^17–19^ define outcome measurements;^20^ assess research priorities;^21^ confirm diagnostic quality indicators and assessment guidelines;^22^ guide the development of electronic patient records;^23^ define registry data collection criteria;^6^ validate prognostic models;^24^ and establish disease definitions.^25–29^ Specific examples include the use of consensus studies to generate clinical recommendations on the optimal assessment and management of chronic obstructive pulmonary disease^30^,^31^ and the selection of candidates for lung transplantation,^32^ to inform treatment and research priorities in pediatric acute respiratory distress syndrome,^33^ to guide selection and use of inhaler devices,^17^,^19^ to assist primary care diagnosis of respiratory diseases,^34^ and to create a standardized list of variables for an international severe asthma registry (ISAR).^6^ A notable example of consensus methods providing interim clinical guidance was the modified Delphi approach that generated recommendations for tapering oral corticosteroids in patients with asthma, an aspect of clinical practice traditionally outside the scope of asthma management guidelines.^5^ A panel of 131 international experts contributed to the guidance, which offered valuable support to clinicians while they awaited more definitive, empirical evidence^35^ and formal guidelines.^36^ The valuable contribution that consensus methods can offer pending more definitive evidence was also illustrated by the publication of 335 consensus-based research publications on the coronavirus disease 2019 (COVID-19) pandemic. Perhaps unsurprisingly, given that decision-making in fast-moving areas and rapidly emerging public health crises can be hampered by a paucity of evidence, the COVID-19 publications included an international Delphi method to develop consensus-based clinical practice statements on the management of COVID-19-related acute respiratory failure; the Delphi process was conducted during the early phase of the pandemic in 2020 and published in 2021.^37^

**Figure 1:**
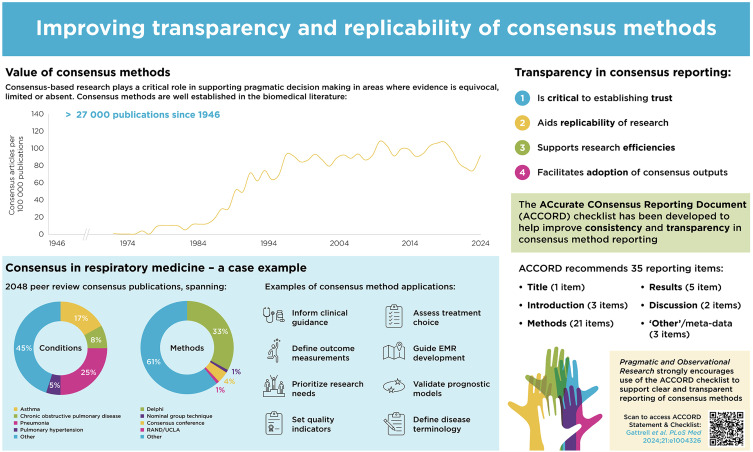
Improving transparency and replicability of consensus methods.

**Notes**: Literature searches conducted via PubMed on July 16, 2024.

**Abbreviation**: EMR, electronic medical record.

The Convergence of Opinion on Recommendations and Evidence (CORE) process is a modified Delphi approach that has been used to support the development of clinical guidelines in respiratory medicine.^8^,^38^ The modifications to the conventional Delphi process were motivated by a desire for greater expediency in guideline development without compromising quality. The process involves the formation of clinical questions and two iterative rounds of an online survey, in which expert panelists vote on the strengths of proposed recommendations, followed by a group discussion. The CORE process was initially validated against eight clinical practice guidelines developed by the American Thoracic Society using conventional consensus approaches. This found that 98% of the CORE-derived recommendations were consistent with the guideline recommendations (based on a threshold of 70% agreement among the experts); however, skepticism of the approach persisted.^8^,^38^ To counter the concerns, the CORE process was further validated using guidelines on idiopathic pulmonary fibrosis and community-acquired pneumonia; again, the CORE process outputs were highly concordant with the guideline recommendations.

The concerns voiced about the CORE process – that it represented a slippery slope and, if used indiscriminately, could lead to inappropriate recommendations – speak to issues of trust and the need for consensus methods to be used appropriately and reported transparently.^39–41^ Indeed, the quality, accuracy, and transparency of reporting of consensus research is important in establishing trust, ensuring the replicability of the consensus methods, and facilitating the adoption of any final recommendations. However, despite the widespread use of consensus methodologies and the important role they play in clinical and policy decision-making, the quality of reporting remains suboptimal, with many studies reported with insufficient details or lacking transparency.^4^

Reporting guidelines have been developed to support the clear and rigorous description of various types of biomedical research, such as the CONsolidated Standards Of Reporting Trials (CONSORT) and Preferred Reporting Items for Systematic Reviews and Meta-Analyses (PRISMA) statements. Until the publication of ACCORD in January 2024, however, there were no comprehensive reporting guidelines for consensus-based research.^1^,^4^ Some publications provided guidance on reporting Delphi methods, but they varied widely in their criteria and level of detail, or were limited in their scope.^42–45^ To address this gap, the ACCORD guideline includes a robust checklist to facilitate the accurate and transparent reporting of all consensus methodologies across all areas of biomedicine.^1^,^3^ Developed through a rigorous Delphi process, the ACCORD checklist comprises 35 recommended reporting items (Table 1), covering the manuscript title (one item), introduction (three items), methods (21 items), results (five items), and discussion (two items). It also includes recommendations for the reporting of “Other” manuscript fields/metadata (three items).^1^,^2^

As of July 2024, the Editor-in-Chief of *Pragmatic and Observational Research* strongly encourages the use of the ACCORD checklist when submitting results of a modified Delphi consensus. Use of the ACCORD checklist^1^ (and explanation and elaboration document^2^) when reporting the results of consensus-based research will help readers to understand the methods used and to interpret the results, thereby contributing to improved transparency and trust in consensus-based research.

**Table 1:** The ACCORD Checklist for Reporting Consensus methods^1^

**Item no.**	**Manuscript Section**	**Item Wording** *Help text*^a^	**Page no.**
T1	Title	Identify the article as reporting a consensus exercise and state the consensus methods used in the title. *For example, Delphi or nominal group technique.*	
I1	Introduction	Explain why a consensus exercise was chosen over other approaches.	
I2		State the aim of the consensus exercise, including its intended audience and geographical scope (national, regional, global).	
I3		If the consensus exercise is an update of an existing document, state why an update is needed, and provide the citation for the original document.	
M1	Methods	If the study or study protocol was prospectively registered, state the registration platform and provide a link. If the exercise was not registered, this should be stated. *Recommended to include the date of registration.*	
M2		Describe the role(s) and areas of expertise or experience of those directing the consensus exercise. *For example, whether the project was led by a chair, co-chairs or a steering committee, and, if so, how they were chosen. List their names if appropriate, and whether there were any subgroups for individual steps in the process.*	
M3		Explain the criteria for panellist inclusion and the rationale for panellist numbers. State who was responsible for panellist selection.	
M4		Describe the recruitment process (how panellists were invited to participate). *Include communication/advertisement method(s) and locations, numbers of invitations sent, and whether there was centralised oversight of invitations or if panellists were asked/allowed to suggest other members of the panel.*	
**Item no.**	**Manuscript Section**	**Item Wording** *Help text*^a^	**Page no.**
M5		Describe the role of any members of the public, patients or carers in the different steps of the study.	
M6		Describe how information was obtained prior to generating items or other materials used during the consensus exercise. *This might include a literature review, interviews, surveys, or another process*.	
M7		Describe any systematic literature search in detail, including the search strategy and dates of search or the citation if published already. *Provide the details suggested by the reporting guideline PRISMA and the related PRISMA-Search extension.*	
M8		Describe how any existing scientific evidence was summarised and if this evidence was provided to the panellists.	
M9		Describe the methods used and steps taken to gather panellist input and reach consensus (for example, Delphi, RAND/UCLA, nominal group technique). *If modifications were made to the method in its original form, provide a detailed explanation of how the method was adjusted and why this was necessary for the purpose of your consensus-based study.*	
M10		Describe how each question or statement was presented and the response options. State whether panellists were able to or required to explain their responses, and whether they could propose new items. *Where possible, present the questionnaire or list of statements as supplementary material.*	
M11		State the objective of each consensus step. *A step could be a consensus meeting, a discussion or interview session, or a Delphi round.*	
M12		State the definition of consensus (for example, number, percentage, or categorical rating, such as “agree” or “strongly agree”) and explain the rationale for that definition.	
M13		State whether items that met the prespecified definition of consensus were included in any subsequent voting rounds.	
M14		For each step, describe how responses were collected, and whether responses were collected in a group setting or individually.	
M15		Describe how responses were processed and/or synthesised. *Include qualitative analyses of free-text responses (for example, thematic, content or cluster analysis) and/or quantitative analytical methods, if used.*	
M16		Describe any piloting of the study materials and/or survey instruments. *Include how many individuals piloted the study materials, the rationale for the selection of those individuals, any changes made as a result and whether their responses were used in the calculation of the final consensus. If no pilot was conducted, this should be stated.*	
M17		If applicable, describe how feedback was provided to panellists at the end of each consensus step or meeting. *State whether feedback was quantitative (for example, approval rates per topic/item) and/or qualitative (for example, comments, or lists of approved items), and whether it was anonymised*.	
M18		State whether anonymity was planned in the study design. Explain where and to whom it was applied and what methods were used to guarantee anonymity.	
M19		State if the steering committee was involved in the decisions made by the consensus panel. *For example, whether the steering committee or those managing consensus also had voting rights.*	
M20		Describe any incentives used to encourage responses or participation in the consensus process. *For example, were invitations to participate reiterated, or were participants reimbursed for their time.*	
M21		Describe any adaptations to make the surveys/meetings more accessible. *For example, the languages in which the surveys/meetings were conducted and whether translations or plain language summaries were available.*	
**Item no.**	**Manuscript Section**	**Item Wording** *Help text*^a^	**Page no.**
R1	Results	State when the consensus exercise was conducted. List the date of initiation and the time taken to complete each consensus step, analysis, and any extensions or delays in the analysis.	
R2		Explain any deviations from the study protocol, and why these were necessary. *For example, addition of panel members during the exercise, number of consensus steps, stopping criteria; report the step(s) in which this occurred.*	
R3		For each step, report quantitative (number of panellists, response rate) and qualitative (relevant socio-demographics) data to describe the participating panellists.	
R4		Report the final outcome of the consensus process as qualitative (for example, aggregated themes from comments) and/or quantitative (for example, summary statistics, score means, medians and/or ranges) data.	
R5		List any items or topics that were modified or removed during the consensus process. Include why and when in the process they were modified or removed.	
D1	Discussion	Discuss the methodological strengths and limitations of the consensus exercise. *Include factors that may have impacted the decisions (for example, response rates, representativeness of the panel, potential for feedback during consensus to bias responses, potential impact of any non-anonymised interactions).*	
D2		Discuss whether the recommendations are consistent with any pre-existing literature and, if not, propose reasons why this process may have arrived at alternative conclusions.	
O1	Other information	List any endorsing organisations involved and their role.	
O2		State any potential conflicts of interests, including among those directing the consensus study and panellists. Describe how conflicts of interest were managed.	
O3		State any funding received and the role of the funder. *Specify, for example, any funder involvement in the study concept/design, participation in the steering committee, conducting the consensus process, funding of any medical writing support. This could be disclosed in the methods or in the relevant transparency section of the manuscript. Where a funder did not play a role in the process or influence the decisions reached, this should be specified.*	

**Notes**: ^a^The ACCORD explanation and elaboration provides guidance and examples to support reporting.^2^ Checklist from: Gattrell WT, Logullo P, van Zuuren EJ, et al. ACCORD (ACcurate COnsensus Reporting Document): a reporting guideline for consensus methods in biomedicine developed via a modified Delphi. *PLoS Med*. 2024;21(1):e1004326.

**Abbreviations**: ACCORD, ACcurate COnsesus Reporting Document; n/a, not applicable; PRISMA, Preferred Reporting Items for Systematic Reviews and Meta-Analyses.

## References

[1] Gattrell WT, Logullo P, van Zuuren EJ, et al. ACCORD (ACcurate COnsensus Reporting Document): a reporting guideline for consensus methods in biomedicine developed via a modified Delphi. *PLoS Med*. 2024;21(1):e1004326. doi:10.1371/journal.pmed.1004326 38261576 PMC10805282

[2] Logullo P, van Zuuren EJ, Winchester CC, et al. ACcurate COnsensus Reporting Document (ACCORD) explanation and elaboration: guidance and examples to support reporting consensus methods. *PLoS Med*. 2024;21(5):e1004390. doi:10.1371/journal.pmed.1004390 38709851 PMC11198995

[3] Gattrell WT, Hungin AP, Price A, et al. ACCORD guideline for reporting consensus-based methods in biomedical research and clinical practice: a study protocol. *Res Integr Peer Rev*. 2022;7(1):3. doi:10.1186/s41073-022-00122-0 35672782 PMC9171734

[4] van Zuuren EJ, Logullo P, Price A, Fedorowicz Z, Hughes EL, Gattrell WT. Existing guidance on reporting of consensus methodology: a systematic review to inform ACCORD guideline development. *BMJ Open*. 2022;12(9):e065154. doi:10.1136/bmjopen-2022-065154 PMC9462098 36201247

[5] Suehs CM, Menzies-Gow A, Price D, et al. Expert consensus on the tapering of oral corticosteroids for the treatment of asthma. A delphi study. *Am J Respir Crit Care Med*. 2021;203(7):871–881. doi:10.1164/rccm.202007-2721OC 33112646

[6] Bulathsinhala L, Eleangovan N, Heaney LG, et al. Development of the International Severe Asthma Registry (ISAR): a Modified Delphi Study. *J Allergy Clin Immunol Pract*. 2019;7(2):578–588.e572. doi:10.1016/j.jaip.2018.08.016 30179741

[7] Murphy MK, Black NA, Lamping DL, et al. Consensus development methods, and their use in clinical guideline development. *Health Technol Assess*. 1998;2(3):i–iv,1–88. doi:10.3310/hta2030 9561895

[8] Wilson KC. Consensus-based recommendations in respiratory medicine. *Eur Respir J*. 2020;56:2002889. doi:10.1183/13993003.02889-2020

[9] Linstone HA TMe. The Delphi method. Techniques and applications; 2002. Available from: https://www.foresight.pl/assets/downloads/publications/Turoff_Linstone.pdf. Accessed June 18, 2024.

[10] Diamond IR, Grant RC, Feldman BM, et al. Defining consensus: a systematic review recommends methodologic criteria for reporting of Delphi studies. *J Clin Epidemiol*. 2014;67(4):401–409. doi:10.1016/j.jclinepi.2013.12.002 24581294

[11] Delbecq AL, van de Ven AH, Gustafson DH. *Group Techniques for Program Planning: A Guide to Nominal Group and Delphi Processes*. Glenview, Illinois, USA: Scott, Foresman and Company; 1975.

[12] Fitch K, Bernstein SJ, Dolores Aguilar M, et al. The RAND/UCLA appropriateness method user’s manual. Santa Monica, California, USA: RAND Corporation; 2001. Available from: https://www.rand.org/pubs/monograph_reports/MR1269.html. Accessed June 18, 2024.

[13] van Melick N, van Cingel RE, Brooijmans F, et al. Evidence-based clinical practice update: practice guidelines for anterior cruciate ligament rehabilitation based on a systematic review and multidisciplinary consensus. *Br J Sports Med*. 2016;50(24):1506–1515. doi:10.1136/bjsports-2015-095898 27539507

[14] Organization WH. The top 10 causes of death; 2020. Available from: https://www.who.int/news-room/fact-sheets/detail/the-top-10-causes-of-death. Accessed June 20, 2024.

[15] Valentine SL, Kudchadkar SR, Ward S, et al. Nonpulmonary treatments for pediatric acute respiratory distress syndrome: from the Second Pediatric Acute Lung Injury Consensus Conference. *Pediatr Crit Care Med*. 2023;24(12 Suppl 2):S45–S60. doi:10.1097/PCC.0000000000003158 36661435

[16] Sweet DG, Carnielli VP, Greisen G, et al. European consensus guidelines on the management of respiratory distress syndrome: 2022 update. *Neonatology*. 2023;120(1):3–23. doi:10.1159/000528914 36863329 PMC10064400

[17] Bousquet J, Winchester C, Papi A, et al. Inhaled corticosteroid/long-acting beta(2)-agonist combination therapy for asthma: attitudes of specialists in Europe. *Int Arch Allergy Immunol*. 2012;157(3):303–310. doi:10.1159/000329519 22056555

[18] Carroll CL, Napolitano N, Pons-Odena M, et al. Noninvasive respiratory support for pediatric acute respiratory distress syndrome: from the second pediatric acute lung injury consensus conference. *Pediatr Crit Care Med*. 2023;24(12 Suppl 2):S135–S147. doi:10.1097/PCC.0000000000003165 36661442

[19] Laube BL, Janssens HM, de Jongh FH, et al. What the pulmonary specialist should know about the new inhalation therapies. *Eur Respir J*. 2011;37(6):1308–1331. doi:10.1183/09031936.00166410 21310878

[20] Needham DM, Sepulveda KA, Dinglas VD, et al. Core outcome measures for clinical research in acute respiratory failure survivors. An international modified Delphi consensus study. *Am J Respir Crit Care Med*. 2017;196(9):1122–1130. doi:10.1164/rccm.201702-0372OC 28537429 PMC5694837

[21] Kelly CA, Kirkcaldy AJ, Pilkington M, et al. Research priorities for respiratory nursing: a UK-wide Delphi study. *ERJ Open Res*. 2018;4(2):00003–02018. doi:10.1183/23120541.00003-2018 29692999 PMC5909062

[22] Hansen MP, Bjerrum L, Gahrn-Hansen B, Jarbol DE. Quality indicators for diagnosis and treatment of respiratory tract infections in general practice: a modified Delphi study. *Scand J Prim Health Care*. 2010;28(1):4–11. doi:10.3109/02813431003602724 20331386 PMC3440613

[23] van Steenkiste BC, Jacobs JE, Verheijen NM, Levelink JH, Bottema BJ. A Delphi technique as a method for selecting the content of an electronic patient record for asthma. *Int J Med Inform*. 2002;65(1):7–16. doi:10.1016/S1386-5056(01)00223-4 11904244

[24] van Royen FS, Moons KGM, Geersing GJ, van Smeden M. Developing, validating, updating and judging the impact of prognostic models for respiratory diseases. *Eur Respir J*. 2022;60(3):2200250. doi:10.1183/13993003.00250-2022 35728976

[25] Nasa P, Bos LD, Estenssoro E, et al. Consensus statements on the utility of defining ARDS and the utility of past and current definitions of ARDS-protocol for a Delphi study. *BMJ Open*. 2024;14(4):e082986. doi:10.1136/bmjopen-2023-082986 PMC11057280 38670604

[26] Iyer N, Khemani R, Emeriaud G, et al. Methodology of the second pediatric acute lung injury consensus conference. *Pediatr Crit Care Med*. 2023;24(12 Suppl 2):S76–S86. doi:10.1097/PCC.0000000000003160 36661437 PMC11069413

[27] Jaworska J, Komorowska-Piotrowska A, Pomiecko A, et al. Consensus on the application of lung ultrasound in pneumonia and bronchiolitis in children. *Diagnostics (Basel)*. 2020;10(11):935. doi:10.3390/diagnostics10110935 33187099 PMC7697535

[28] Maher TM, Whyte MK, Hoyles RK, et al. Development of a consensus statement for the definition, diagnosis, and treatment of acute exacerbations of idiopathic pulmonary fibrosis using the Delphi technique. *Adv Ther*. 2015;32(10):929–943. doi:10.1007/s12325-015-0249-6 26498943 PMC4635174

[29] Upham JW, Le Lievre C, Jackson DJ, Masoli M, Wechsler ME, Price DB. Defining a Severe Asthma Super-Responder: findings from a Delphi Process. *J Allergy Clin Immunol Pract*. 2021;9(11):3997–4004. doi:10.1016/j.jaip.2021.06.041 34271216

[30] Miravitlles M, Acharya S, Aggarwal B, et al. Clinical concepts for triple therapy use in patients with COPD: a Delphi consensus. *Int J Chron Obstruct Pulmon Dis*. 2023;18:1853–1866. doi:10.2147/COPD.S424128 37662490 PMC10474219

[31] Siafakas NM, Vermeire P, Pride NB, et al. Optimal assessment and management of chronic obstructive pulmonary disease (COPD). The European Respiratory Society Task Force. *Eur Respir J*. 1995;8(8):1398–1420. 7489808 10.1183/09031936.95.08081398

[32] Weill D, Benden C, Corris PA, et al. A consensus document for the selection of lung transplant candidates: 2014--an update from the pulmonary transplantation council of the international society for heart and lung transplantation. *J Heart Lung Transplant*. 2015;34(1):1–15. doi:10.1016/j.healun.2014.06.014 25085497

[33] Group PALICC. Pediatric acute respiratory distress syndrome: consensus recommendations from the Pediatric Acute Lung Injury Consensus Conference. *Pediatr Crit Care Med*. 2015;16(5):428–439. doi:10.1097/PCC.0000000000000350 25647235 PMC5253180

[34] Levy ML, Fletcher M, Price DB, Hausen T, Halbert RJ, Yawn BP. International Primary Care Respiratory Group (IPCRG) guidelines: diagnosis of respiratory diseases in primary care. *Prim Care Respir J*. 2006;15(1):20–34. doi:10.1016/j.pcrj.2005.10.004 16701756 PMC6730677

[35] Menzies-Gow A, Gurnell M, Heaney LG, et al. Oral corticosteroid elimination via a personalised reduction algorithm in adults with severe, eosinophilic asthma treated with benralizumab (PONENTE): a multicentre, open-label, single-arm study. *Lancet Respir Med*. 2022;10(1):47–58. doi:10.1016/S2213-2600(21)00352-0 34619104

[36] Papadopoulos NG, Custovic A, Deschildre A, et al. Recommendations for asthma monitoring in children: a PeARL document endorsed by APAPARI, EAACI, INTERASMA, REG, and WAO. *Pediatr Allergy Immunol*. 2024;35(4):e14129. doi:10.1111/pai.14129 38664926

[37] Nasa P, Azoulay E, Khanna AK, et al. Expert consensus statements for the management of COVID-19-related acute respiratory failure using a Delphi method. *Crit Care*. 2021;25(1):106. doi:10.1186/s13054-021-03491-y 33726819 PMC7962430

[38] Schoenberg NC, Barker AF, Bernardo J, et al. A comparative analysis of pulmonary and critical care medicine guideline development methodologies. *Am J Respir Crit Care Med*. 2017;196(5):621–627. doi:10.1164/rccm.201705-0926OC 28731387 PMC5955064

[39] Dahm P, Sultan S, Murad MH. A blast from the past-back to the 1970s. *Am J Respir Crit Care Med*. 2018;197(11):1500–1501. doi:10.1164/rccm.201711-2186LE 29345971

[40] Schunemann HJ, Brozek JL. No room for error in medicine-A case of deja vu. *Am J Respir Crit Care Med*. 2018;197(11):1501–1502. doi:10.1164/rccm.201710-2076LE 29345966

[41] Shah R, Morgan RL, Falck-Ytter Y, Mustafa RA. Have we not learned from past mistakes? *Am J Respir Crit Care Med*. 2018;197(11):1499–1500. doi:10.1164/rccm.201711-2200LE 29345959

[42] Brouwers MC, Kerkvliet K, Spithoff K, Consortium ANS. The AGREE reporting checklist: a tool to improve reporting of clinical practice guidelines. *BMJ*. 2016;352:i1152. doi:10.1136/bmj.i1152 26957104 PMC5118873

[43] Junger S, Payne SA, Brine J, Radbruch L, Brearley SG. Guidance on Conducting and REporting DElphi Studies (CREDES) in palliative care: recommendations based on a methodological systematic review. *Palliat Med*. 2017;31(8):684–706. doi:10.1177/0269216317690685 28190381

[44] Kirkham JJ, Gorst S, Altman DG, et al. Core outcome set-standards for reporting: the COS-STAR statement. *PLoS Med*. 2016;13(10):e1002148. doi:10.1371/journal.pmed.1002148 27755541 PMC5068732

[45] Spranger J, Homberg A, Sonnberger M, Niederberger M. Reporting guidelines for Delphi techniques in health sciences: a methodological review. *Z Evid Fortbild Qual Gesundhwes*. 2022;172:1–11. doi:10.1016/j.zefq.2022.04.025 35718726

